# Supplementary material for: Fecal Carriage of Extended-Spectrum β-Lactamase-/AmpC-Producing Escherichia coli in Pet and Stray Cats
Source: Antibiotics (Basel). 2023 Jul 29;12(8):1249. doi: 10.3390/antibiotics12081249 (PMC10451524; doi:10.3390/antibiotics12081249)
Supplement: Supplementary file 1 [file antibiotics-12-01249-s001.zip › antibiotics-2517779-supplementary.pdf]

Table S1. Characteristics of unhealthy cats compared to healthy cats analyzed in this study.

Characteristics of cats		Total cats (no. 97)	Unhealthy cats (no. 27)	Healthy cats (no. 70)
Sex	Male n. (%)	54 (55.7)	17 (63)	37 (52.9)
	Female n. (%)	43 (44.3)	10 (37)	33 (47.1)
Age	<2 years n. (%)	55 (56.7)	12 (44.4)	43 (61.4)
	≥2 years n. (%)	40 (41.2)	14 (51.9)	26 (37.1)
	ND (%)	2 (3.1)	1 (3.7)	1 (1.4)
Type of ownership	Stray n. (%)	50 (51.5)	0 (0)	50 (71.4)
	Owned n. (%)	47 (48.5)	27 (100)	20 (28.6)
Hospitalization	Yes n. (%)	10 (10.3)	10 (37)	0 (0)
	No n. (%)	87 (89.7)	17 (63)	70 (100)
Previous antibiotic therapy	Yes n. (%)	25 (25.8)	18 (66.7)	7 (10)
	No n. (%)	72 (74.2)	9 (33.3)	63 (90)

Table S2. Results of the chromosomal ampC promoter/ attenuator regions analysis of the AmpC-producing *E. coli* detected in this study.

Position within the *ampC* promoter/attenuator regions[1]		ATCC 25922 (accession number: CP009072)	46/1 (AmpC-producing *E. coli* detected in this study)
Displaced -35 box	-42	C	
-35 box	-32	T	A
	-28	G	A
	-26.1	-	
	-23	T	
	-20.1	-	
Displaced -10 box	-18	G	
Spacer	-15.2	-	
	-15.1	-	
	-15	G	
-10 box	-11	C	
Attenuator	+17	C	
	+21.1	-	
	+22	C	
	+23	G	
	+24	C	
	+26	T	
	+27	A	
	+28	T	
	+30	G	
	+32	G	
	+34	G	
	+35	C	
	+36	C	
	+37	G	

[1]Descriptions of functional elements are used as reported by Peter-Getzlaff et al., 2011 and Coolen et al., 2021 [20,60].

Table S3. Primers sequences used in this study.

Target	Primer	Sequence 5′-3′	Reference
CTX-M	*bla*$_{CTX-M}$ F	ATGTGCAGYACCAGTAARGTKATGGC	[51]
	bla$_{CTX-M}$ R	TGGGTRAARTARGTSACCAGAAYCAGCGG	[51]
TEM	*bla*$_{TEM}$ F	TCGCCGCATACACTATTCTCAGAATGA	[51]
	bla$_{TEM}$ R	ACGCTCACCGGCTCCAGATTTAT	[51]
SHV	*bla*$_{SHV}$ F	ATGCGTTATATTCGCCTGTG	[51]
	bla$_{SHV}$ R	TGCTTTGTTATTCGGGCCAA	[51]
CTX-M-1 group	*bla*$_{CTX-M-1}$ F	GGTTAAAAAATCACTGCGYC	[52]
	bla$_{CTX-M-1}$ R	TYGGTGACGATTTTAGCCGC	[52]
CTX-M-2 group	*bla*$_{CTX-M-2}$ F	ATGATGACTCAGAGCATTCG	[53]
	bla$_{CTX-M-2}$ R	TCAGAAACCGTGGGTTAC	[53]
CTX-M-8 group	*bla*$_{CTX-M-8}$ F	TGATGAGACATCGCGTTAAG	[54]
	bla$_{CTX-M-8}$ R	TAACCGTCGGTGACGATTTT	[54]
CTX-M-9 group	*bla*$_{CTX-M-9}$ F	ATGGTGACAAAGAGAGTGCA	[55]
	bla$_{CTX-M-9}$ R	CCCTTCGGCGATGATTCTC	[55]
CTX-M-25 group	*bla*$_{CTX-M-25}$ F	CACACGAATTGAATGTTCAG	[56]
	bla$_{CTX-M-25}$ R	TCACTCCACATGGTGAGT	[56]
ACC-1 and ACC-2	MultiCaseACC_F	CACCTCCAGCGACTTGTTAC	[58]
	MultiCaseACC_R	GTTAGCCAGCATCACGATCC	[58]
FOX-1 to FOX-5	MultiCaseFOX_F	CTACAGTGCGGGTGGTTT	[58]
	MultiCaseFOX_R	CTATTTGCGGCCAGGTGA	[58]
MOX-1, MOX-2, CMY-1, CMY-8 to CMY-11 and CMY-19	MultiCaseMOX_F	GCAACAACGACAATCCATCCT	[58]
	MultiCaseMOX_R	GGGATAGGCGTAACTCTCCCAA	[58]
DHA-1 and DHA-2	MultiCaseDHA_F	TGATGGCACAGCAGGATATTC	[58]
	MultiCaseDHA_R	GCTTTGACTCTTTCGGTATTCG	[58]
LAT-1 to LAT-3, BIL-1, CMY-2 to CMY-7, CMY-12 to CMY-18 and CMY-21 to CMY-23	MultiCaseCIT_F	CGAAGAGGCAATGACCAGAC	[58]
	MultiCaseCIT_R	ACGGACAGGGTTAGGATAGY	[58]
ACT-1 and MIR-1	MultiCaseEBC_F	CGGTAAAGCCGATGTTGCG	[58]
	MultiCaseEBC_R	AGCCTAACCCCTGATACA	[58]
campC promoter/attenuator region	AmpC AB1	GATCGTTCTGCCGCTGTG	[59]
	AmpC-2	GGGCAGCAAATGTGGAGCAA	[59]
chuA	chuA.1b	ATGGTACCGGACGAACCAAC	[44]
	chuA.2	TGCCGCCAGTACCAAAGACA	[44]
yjaA	yjaA.1b	CAAACGTGAAGTGTCAGGAG	[44]
	yjaA.2b	AATGCGTTCCTCAACCTGTG	[44]
TspE4.C2	TspE4C2.1b	CACTATTCGTAAGGTCATCC	[44]
	TspE4C2.2b	AGTTTATCGCTGCGGGTCGC	[44]
arpA	AceK.f	AACGCTATTCGCCAGCTTGC	[44]
	ArpA1.r	TCTCCCCATACCGTACGCTA	[44]

Table S4. List of antimicrobials used, related cut-off values and the range of MIC (mg/L) used in this study.

Antimicrobial class	Antimicrobial agent	Cut-off (μg/mL)*	range of MIC (μg/mL)
β-Lactam (penicillins)	Ampicillin (AMP)	>8	0.25-8
β-Lactam (Cephalosporin I)	Cefazolin (FAZ)	≥32	1-32
	Cephalexin (LEX)	≥32	0.5-16
β-Lactam (Cephalosporin III)	Cefovecin (FOV)	≥8	0.25-8
	Cefpodoxime (POD)	≥8	1-8
	Ceftazidime (TAZ)	≥16	4-16
β-Lactams and β-Lactamase Inhibitors	Amoxicillin/clavulanic acid 2:1 (AUG2)	>8/4	0.25/0.12-8/4
	Piperacillin/tazobactam contant 4 (P/T4)	≥32/4	8/4-64/4
Aminoglycosides	Amikacin (AMI)	≥16	4-32
	Gentamicin (GEN)	≥8	0.25-8
Phenicol	Chloramphenicol (CHL)	≥32	2-32
Fluoroquinolones	Enrofloxacin (ENRO)	≥4	0.12-4
	Marbofloxacin (MAR)	≥4	0.12-4
	Orbifloxacin (ORB)	≥8	1-8
	Pradofloxacin (PRA)	≥2	0.25-2
Tetracyclines	Tetracycline (TET)	≥16	4-16
	Doxycycline (DOX)	≥0.5	0.25-8
Carbapenems	Imipenem (IMI)	≥4	1-8
Folate pathway antagonists	Trimethoprim/sulfamethoxazole 1:19 (SXT)	≥4/72	0.5/9.5-4/72

* CLSI VET01S ED6:2023 cutoff values [61].
